# Stresses induced by one piece and two piece dental implants in All-on-4® implant supported prosthesis under simulated lateral occlusal loading: non linear finite element analysis study

**DOI:** 10.1186/s12903-022-02228-9

**Published:** 2022-05-22

**Authors:** Ahmed Mostafa Abdelfattah Mohamed, Mohamed Gamal Askar, Mahmoud El-Moutassim Bellah El Homossany

**Affiliations:** 1 Oral and Maxilofacial Prosthodontics Department, Faculty of Dentistry, AinShams University, Organization of African Unity Street, Cairo, 11561 Egypt; 2 Mechanical Department, Faculty of Engineering, Helwan University, Cairo, Egypt

**Keywords:** One piece dental implant, Two piece dental implant, Group function occlusion, Canine guided occlusion, All-on-4®, Non linear finite element analysis

## Abstract

**Background:**

Correct choice of the implant design and the occlusal scheme is important for the success of implant supported restorations. So, the aim of the current study was to find out the difference in the stresses induced by the one piece dental implants designed to be used in the All-on-4® concept and the conventional two piece ones under simulated lateral occlusal schemes using nonlinear finite element analysis.

**Methods:**

Two finite element models of the maxilla, implants, and prostheses were designed according to the All-on-4® concept. In the model TP, two piece dental implants were placed while in the model OP one piece dental implants were used. Two loading scenarios were applied to each model; the first one simulated a group function occlusal scheme while the second scenario simulated a canine guided one.

**Results:**

The highest stress value was recorded in the model TP with the group function occlusion and the lowest stress value was in the model OP with the canine guidance occlusion.

**Conclusion:**

The one-piece dental implants can be concluded to induce less stress compared to the two piece dental implants when used in the All-on-4® implant supported prosthesis in the different lateral occlusal schemes. Canine guided occlusion can be concluded to cause lower stress values in comparison to the group function occlusal scheme.

## Background

Dental implants offer a valid treatment option for rehabilitation of the edentulous arches. However, the maxillary arch is challenging for dental implant placement. This could be attributed to the pattern of bone resorption and sinus pneumatization. Although bone grafting and sinus floor elevation can solve such problems yet they offer some disadvantages as being complex surgical procedures, having added cost and questionable predictability [1].

The All-on-four concept was introduced as a treatment modality for the rehabilitation of the atrophic edentulous maxilla using dental implants to overcome the previous disadvantages. Although the presence of additional implants in the All-on-six implant supported prosthesis helped to decrease the recorded stress values compared to the All-on-4® one yet, they had similar patterns of stress distribution and location as stated by Behring et al. [2] and Silva et al. [3] in their Finite Element studies. Behring et al. [2] also concluded that the biomechanical behavior of the All-on-4® concept becomes improved with increasing the rigidity of the implant framework. Furthermore, Ayali et al. [4] reported no great differences in the maximum stress values between the different All-on-4® configurations as the M-4 and the V-4. The narrow and wide implant diameters showed a comparable biomechanical behavior when being used in the All-on-4® concept [5]. Ozan et al. [6] also stated that tilting the posterior implants helped to reduce the cantilever length and decrease the stress values in the periimplant bone and screws. Moreover, the clinical studies showed high success rate for such a treatment modality [7, 8]. On the other hand, biological complications as periimplant mucositis and reduced marginal bone levels were mentioned in the literature. Mechanical complications as occlusal material chipping and screw loosening or fracture in the implant supported fixed restorations were also reported [9, 10]. However, long term success rate is affected by number of factors as the design of the implant abutment complex and the occlusal scheme of the overlying prostheses [11].

Ideal design of the implant abutment complex should allow proper load distribution to be delivered within the physiological tolerance of the supporting structures. The implant-abutment connection design also affects the implant survival rate, peri-implant bone resorption and frequency of screw loosening [12]. However, different designs are commercially available as the one piece and two piece dental implants. The presence of microgaps between the implant fixture and the overlying abutment in the two piece dental implants was found to be associated with microleakage and bacterial contamination. Such a reason was used to justify the increased levels of periimplant marginal bone loss in the two piece dental implants compared to the one piece ones [13]. Moreover, Finite Element Analysis showed higher stress values recorded in the crestal bone surrounding the two piece dental implants compared to the one piece ones. Such higher stress values were also suggested to be a cause for the increased level of marginal bone loss in the two piece dental implants [14, 15]. On the other hand, Vorous et al. [16] stated non significant difference in their systematic review between both implant types regarding the level of marginal bone loss. Furthermore, Liu et al. [17] concluded in their subgroup meta analysis that the two piece dental implants with platform switching had a significant reduced level of marginal bone loss compared to the one piece ones.

Screw loosening was among the most frequently reported complications in the implant supported fixed restorations [10, 12, 18]. Screw loosening causes micromotion of the overlying prosthesis and consequent soft tissue irritation [19]. Furthermore, Ragauskite et al. stated that the occlusal material cracking in the screw retained prosthesis occurred secondary to the screw loosening [20]. Meanwhile, as a result of screw absence in the one piece dental implant design, the risk of screw loosening or fracture is eliminated; a frequent complication that occurs with the two piece dental implants [21–23]. On the other hand, further studies comparing the different outcomes between the one piece and the two piece dental implants showed that one piece dental implants had a higher rate of screw loosening compared to the two piece ones. Such a result was attributed to the use of an intermediate abutment between the one piece dental implant and the prosthetic superstructure [24, 25].

Proper selection of the occlusal scheme is also an important factor for the success and longevity of the implant supported prosthesis. Occlusal overload was reported to be highly related with implant failure [26, 27]. Group functional occlusion and canine guided occlusion are concepts that were proposed to be used with implant supported fixed prosthesis. It was reported that the group functional occlusion was associated with increased levels of marginal bone loss in the implant supported fixed prosthesis compared with the canine guided occlusal scheme. This was related to the great occlusal forces generated during the lateral mandibular excursions. Further reasons included the angle of the occlusal forces, the possibility of contact with the opposing teeth in non-functional lateral movements and possible non-working side contacts in the group function design [28]. On the other hand, in the canine guided occlusion, contact is only present within the canine teeth during the lateral excursions and all other teeth are protected from unnecessary occlusal loads [29]. However, Abdou et al. showed in their systematic review that both occlusal schemes developed similar stresses during maximum intercuspation and the protrusive excursions. While during the lateral excursions the group functional occlusal scheme developed twice the stresses as the canine guided one [30].

The purpose of the current study was to find out the difference in the stresses induced by the one piece dental implants designed to be used in the All-on-4® concept (Bio Art Uno, Flotechno dental implants, Milano, Italy)® and the conventional two piece ones supporting the All-on-4® implant supported prosthesis under simulated lateral occlusal schemes using nonlinear Finite Element Analysis. Such a design of the one piece dental implant in the All-on-4® concept was claimed to reduce the possible complications of the conventional two piece one as the components’ fractures and the risk of abutment unscrewing. The assumed null hypothesis was that no difference existed in the stress values induced by both implant designs when used in the All-on-4® implant supported prosthesis when lateral occlusal loading is simulated.

## Methods

The current study included two steps: virtual model construction and three-dimensional Finite Element Analysis. Two virtual models were made in this study. In the virtual model TP, two-piece dental implants with multiunit abutments were used. However, in the virtual model OP, one-piece dental implants with titanium bases were used.

### Model construction

For virtual model construction, an educational maxillary edentulous cast (Ramses medical products, Cairo, Egypt) was used. It was scanned using 3D scanner (CeraMap 400 Amann Girrbach America Inc., Koblach, Austria) and modelled using CAD/CAM software (Solidworks2020 SolidWorks Corp., Dassault Systèmes, Villacoublay, France). As (D3) bone density is often observed in the maxilla, the cast was virtually formed to represent a 1 mm outer cortical bone covering the trabecular bone. Reverse Engineering was made to the cast and exported in Standard Tessellation Language (STL) file. This STL virtual cast was imported into the Mesh Mixer software (Mesh Mixer, Autodesk, San Rafael, California, United States of America) for further smoothening, gap filling and exported as STL format.

Four dental implants (BioArtUno, Flotechno dental implants, Milano, Italy) were planned to be placed in the lateral incisor and premolar region following the All-on-4® concept. The anterior implants were axially placed whereas the posterior ones were placed with a distal angulation 17° with anteroposterior spread 18 mm [31]. The implants were modelled using the Solidworks software (SolidWorks Corp., Dassault Systèmes, Villacoublay, France) after their dimensions were taken from the user’s manual. Multiunit abutments and titanium bases were also modelled using the Solidworks software.

The prosthetic superstructure was designed using Exocad software (Exocad America, Inc. Darmstadt, Germany) to be in the form of Titanium implant bridge supporting zirconia crowns [32–35]. The STL files of the dental implants, abutments and prosthetic superstructure were imported into the Mesh Mixer software for smoothening and gap filling.

The STL files of the edentulous maxillary cast, dental implants, abutments, and prosthetic superstructure were imported into Siemens NX10 (Unigraphics NX, Siemens PLM, California, USA) for conversion into solid parts. Superimposition of the cortical and cancellous parts of bone was done followed by Boolean subtraction to get the three dimensional virtual maxillary cast. Superimposition and Boolean subtraction were then made for the implants in the virtual casts. Finally, superimposition of the abutments and the prosthetic superstructures were made followed by Boolean subtraction. Parasolid Extension files were then exported and imported into the SolidWorks software for components assembly. Interference was also checked by interference detection tool.

**Fig. 1:**
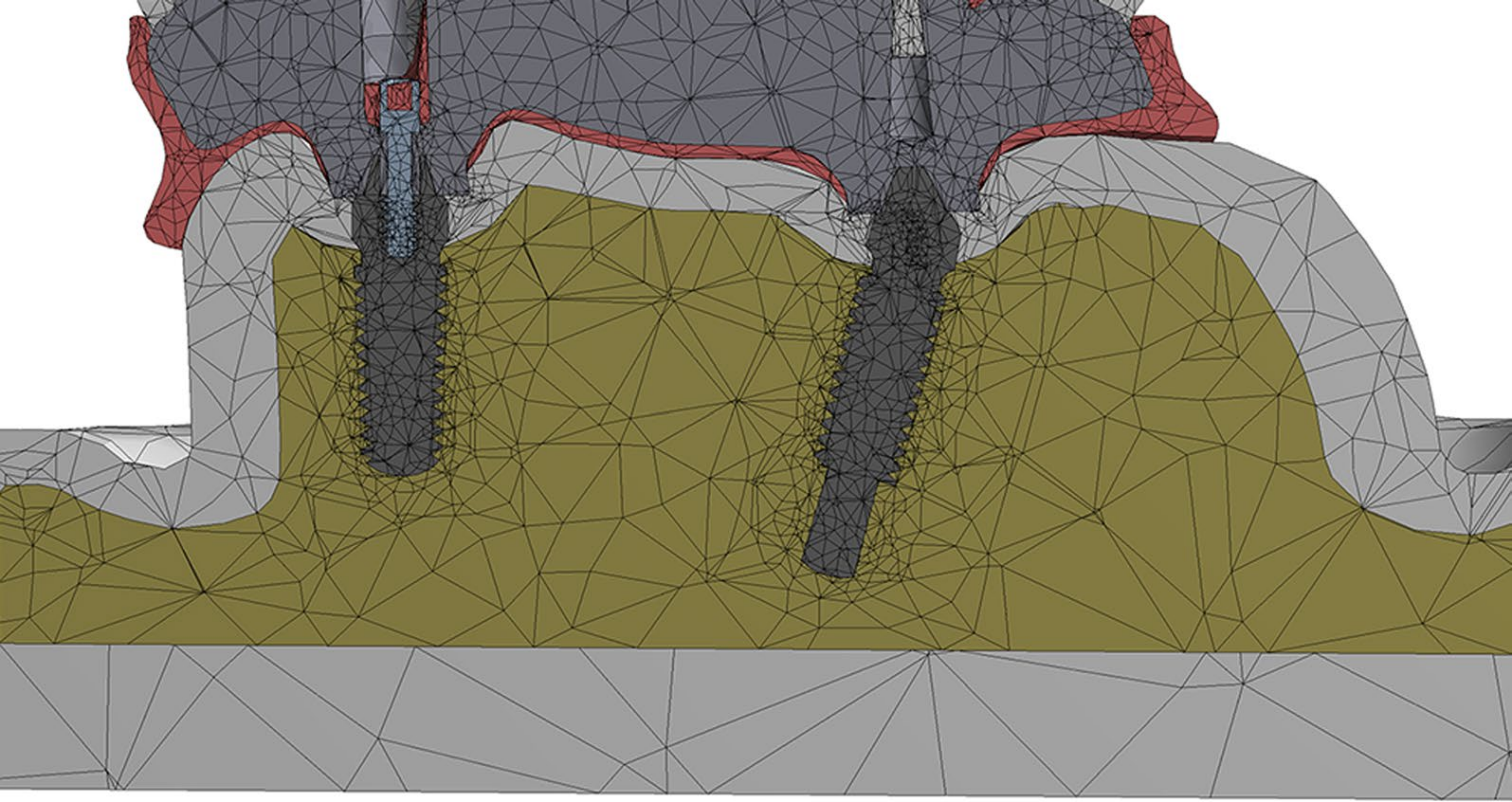
A Cross section in the meshed model OP showing the one piece dental implants in the bone and the overlying superstructure

The components were exported from the Solidworks software into the ANSYS16.2 software (Ansys, Inc, Pennsylvania, USA) program and presented as a function of area. These areas were then converted to volumes (Fig. 1). The parabolic tetrahedral element was the element of choice used. For non linear static analysis, the Elastic Modulus and the Poisson's Ratio were defined for each component. The properties are listed in Table 1 [31–33]. All model materials were isotropic, homogenous, and linearly elastic. Implants were completely osseointegrated with bone. Coefficient of friction (0.2) was given between the implants and prosthetic superstructures for non linear static analysis [5]. The total number of elements in the model TP was 3,474,771 and in the model OP was 167,445. However, the number of nodes in the model TP was 4,952,640 and in the model OP was 302,504.

**Table 1:** Properties of each component in the model [31–33]

Material	Modulus of elasticity (MPa)	Poisson's ratio
Compact bone	13,700	0.3
Cancellous bone	7930	0.3
Zirconia	200,000	0.3
Titanium alloy	110,000	0.33

*MPa* Mega Pascal unit

### Load application

**Fig. 2:**
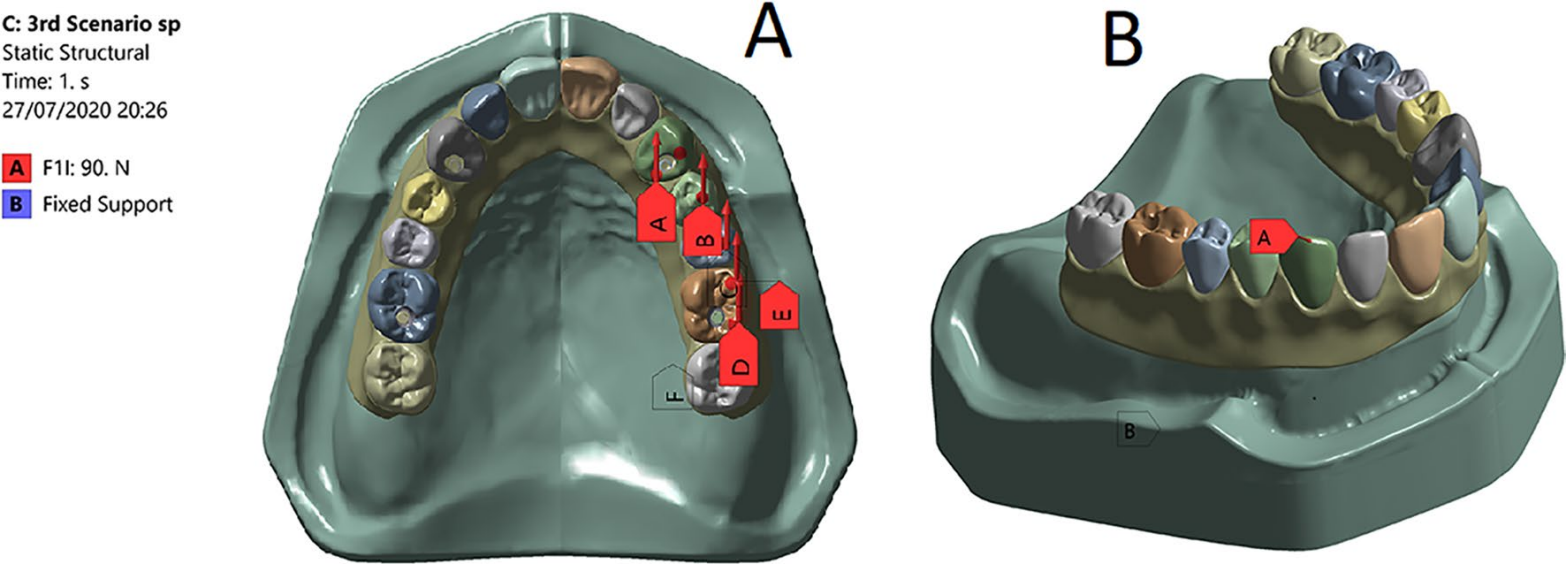
**a** A photo showing the group function loading scenario with a a unilateral 90 N horizontal static load applied to the palatal surface of the canine, the buccal cusp of the first and the second premolars in addition to the mesiobuccal and the distobuccal cusps of the first molar with a total load of 450 N. **b** A photo showing the canine guided loading scenario with a unilateral 90 N horizontal static load applied to the palatal surface of the canine

For load application, a three dimensional Finite Element ball model (5.8 mm in diameter) was used. Two loading scenarios were applied [31, 36]. The first scenario simulated the group function occlusion in which a unilateral 90 N horizontal static load was applied to the palatal surface of the canine, the buccal cusp of the first and the second premolars in addition to the mesiobuccal and the distobuccal cusps of the first molar with a total load 450 N (Fig. 2a). The second scenario simulated the canine guided occlusion in which a unilateral 90 N horizontal static load was applied to the palatal surface of the canine (Fig. 2b).

The boundary condition was defined in such a way that all movements at the base of the virtual model were restrained during load application in all directions. Each model was analyzed with the same exact boundary conditions and load application. The stresses displayed in this study are the maximum principle stress for the bone and the (SEqv) von-Mises stress for the implants and the prosthetic screws [37]. They were displayed as graphical output in the form of color coded maps and numeric output that displayed the amount of the maximum equivalent stresses (Von-Mises stresses) and the maximum principal stress in Megapascal (Mpa). One single investigator calculated the average stress value for the implant abutment complex in both models. Calculation was made by summation of the stress values recorded in each cell of the mesh constituting the component followed by division upon the number of the cells.

## Results

**Fig. 3:**
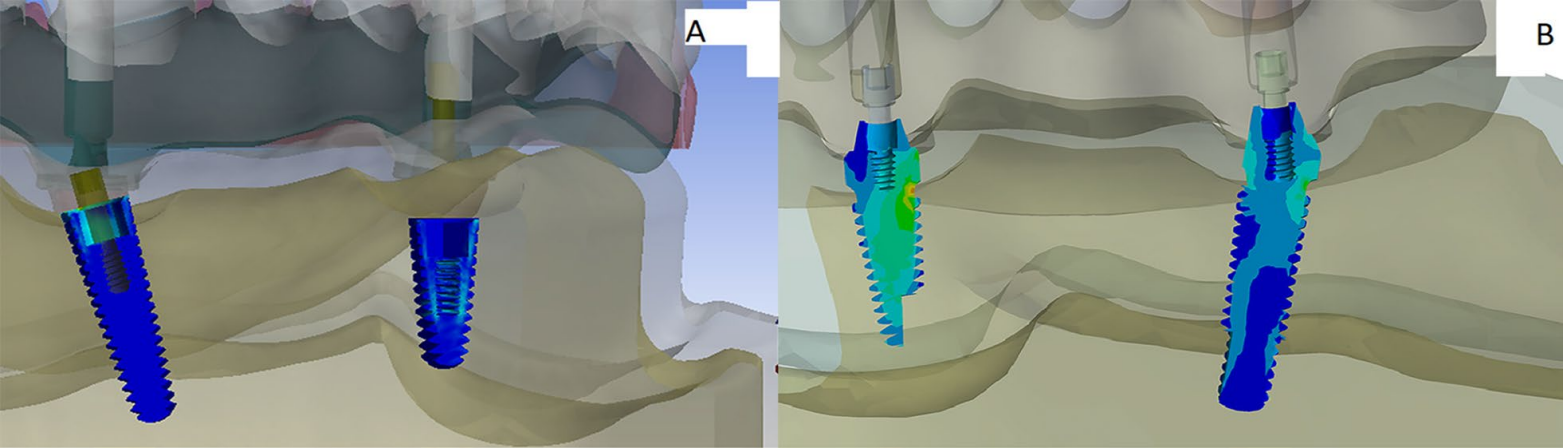
**a** A photo of the stress distribution in the dental implants in the model TP during the group functional and canine guided occlusal schemes showing maximum stress concentration in the coronal part of the dental implants. **b** A photo of the stress distribution in the dental implants in the model OP during the group functional and canine guided occlusal schemes showing maximum stress concentration in the junction of the implant abutment complex

For the stresses induced in the implants, stress concentration was observed in the crestal region of the posterior and the anterior implants on the loaded side in the model TP during both loading scenarios (Fig. 3a). While for the model OP, it was observed in the junction of the implant-abutment complex in the anterior and posterior implants during both loading scenarios (Fig. 3b). The highest stress value (108.3 Mpa) was recorded in the model TP during group functional occlusion while the lowest (4.17 MPa) was recorded in the model OP during canine guided one. However, the maximum value in the model TP was higher than in the model OP for both loading scenarios. Moreover, the stress values recorded in the dental implants for the group function occlusion was higher than the canine guided one for each model. The maximum value of the Von Mises stress in both models for both loading scenarios is listed in Table 2. Moreover, the average stress value for the anterior implant was higher than the posterior implant in the model OP for both loading scenarios. However, in the model TP, the anterior implant had a lesser average stress value compared to the posterior implant. The average stress value of the Von Mises stress in both models for both loading scenarios is listed in Table 3.

**Table 2:** Comparison of the maximum recorded stress values in the implants, screws and bone between the models OP and TP during group functional and canine guided loading scenarios

	Loading scenario	Model TP (MPa)	Model OP (MPa)
Dental implant	Scenario 1: Group functional occlusion	108.3*	18.49*
	Scenario 2: Canine guidance occlusion	14.1*	4.17*
Screw	Scenario 1: Group functional occlusion	59.35*	43.84*
	Scenario 2: Canine guidance occlusion	8.18*	6.71*
Bone	Scenario 1: Group functional occlusion	88.24^#^	16.06^#^
	Scenario 2: Canine guidance occlusion	13.43^#^	2.72^#^

*MPa* Mega Pascal unit

^*^Symbol indicates VonMises stress

^#^Symbol indicates maximum principal stress

**Table 3:** Comparison of the average stress values induced in the implants and screws between the models OP and TP during group functional and canine guided loading scenarios

Model component	Loading scenario	Model TP		Model OP	
		Anterior (MPa)	Posterior (MPa)	Anterior (MPa)	Posterior (MPa)
Dental Implant	Scenario1: Group functional occlusion	6.32	9.47	3.47	2.62
	Scenario2: Canine guidance occlusion	0.85	1.01	0.63	0.53
Screw	Scenario1: Group functional occlusion	4.41	5.84	2.91	2.01
	Scenario2: Canine guidance occlusion	0.68	1.01	0.54	0.46

*MPa* Mega Pascal unit

**Fig. 4:**
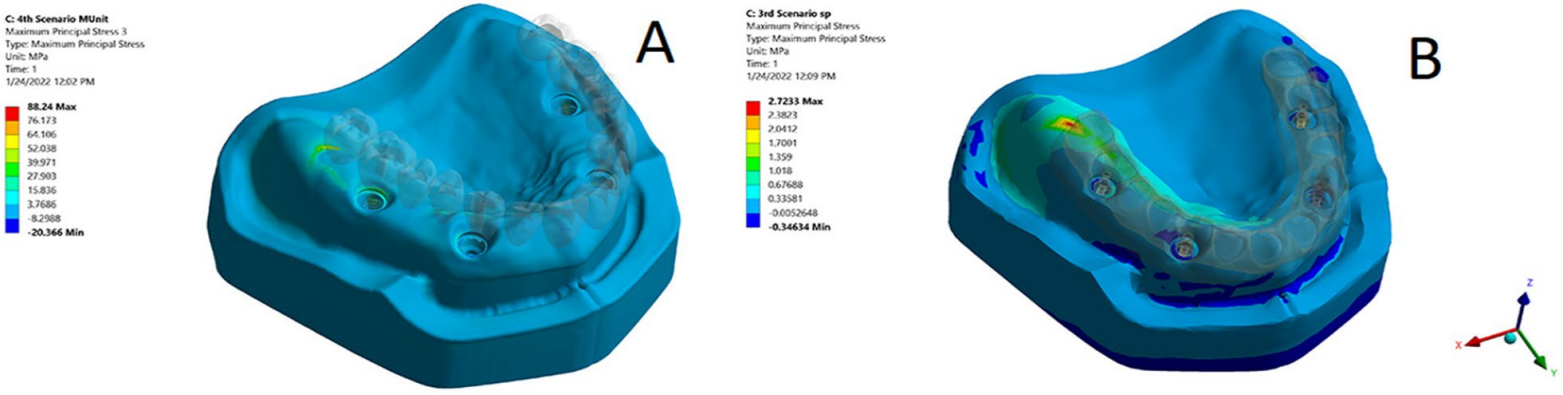
**a** A photo of the stress distribution in the bone in the model TP during the group functional and canine guided occlusal schemes showing maximum stress concentration in the crestal part of the bone. **b** A photo of the stress distribution in the bone in the model OP during the group functional and canine guided occlusal schemes showing maximum stress concentration in the crestal part of the bone

Regarding the stresses induced in the supporting bone, the principal stress was concentrated in the crestal bone surrounding the posterior and anterior implants in the loaded side in both models during both loading scenarios (Fig. 4a, b). However, higher concentrations were observed posteriorly rather than anteriorly. The highest stress value (88.24 Mpa) was recorded in the model TP during group functional occlusion while the lowest (2.72 MPa) was recorded in the model OP during canine guided one. The model TP had higher stress values compared to the model OP during both loading scenarios. Moreover, the stress values recorded in the bone for the group functional occlusion was higher than the canine guided one for each model. The maximum value for the maximal principal stress in both models during both loading scenarios is shown in Table 2.

**Fig. 5:**
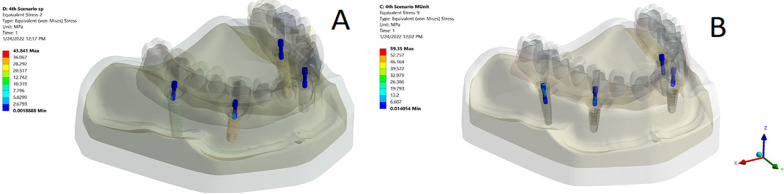
**a** A photo of the stress distribution in the screw in the model TP during the group functional and canine guided occlusal schemes showing maximum stress concentration in the posterior screw. **b** A photo of the stress distribution in the screw in the model OP during the group functional and canine guided occlusal schemes showing maximum stress concentration in the anterior screw

While for the stresses induced in the prosthetic screw, stress concentration was observed in the anterior and posterior screws of the loaded side in both models during both loading scenarios. In both loading scenarios, the posterior screw showed higher stress value compared to the anterior one in the model TP (Fig. 5a) and the anterior screw showed higher stress values compared to the posterior one in the model OP (Fig. 5b). The highest stress value (59.35 Mpa) was recorded in the model TP during group functional occlusion while the lowest (6.71 MPa) was recorded in the model OP during canine guided one. However, the maximum value in the model TP was higher than in the model OP for both loading scenarios. Moreover, the stress values recorded in the prosthetic screw for the group functional occlusion was higher than the canine guided one for each model. The maximum value of the Von Mises stress in both models in both loading scenarios is listed in Table 2 and the average stress values are listed in Table 3.

## Discussion

The purpose of this study was to find out the difference in the stresses induced by the one piece and the two piece dental implants supporting the All-on-4® implant supported prosthesis under simulated lateral occlusal schemes using non linear Finite Element Analysis. The null hypothesis was rejected as the one piece dental implant induced less stress values compared to the two piece dental implant for all simulated scenarios. The canine guidance simulation also resulted in less stress values compared to the group functional one.

The posterior implants were distally inclined 17° in the current study as this inclination induced less stress values compared to the 45° one [31]. Owing to its biocompatibility, low density and favorable mechanical properties, titanium alloy was selected to be the implant bridge material in this study [32]. Zirconia was also selected for its esthetical outcome. Furthermore, stress analysis studies showed comparable results between the different occlusal materials regarding the stress pattern induced by them on the supporting structures in the implant supported fixed restorations [33–35].

The magnitude, distribution and direction of loads used in this study were based on previous studies [31, 36, 38]. Delayed loading was adopted in the current study so, complete bone osseointegration with the dental implants were assumed. For more realistic simulation, nonlinear static analysis was adopted in the implant abutment complex design and the friction coefficient was set to 0.2 [5]. The Von Mises stress values were used in this study to display the results as it is the most commonly used measurement for evaluating the yielding behavior of the materials [37]. The maximum value of the Von Mises stress was recorded in the crestal region of the dental implants on the loaded side in the model TP during both loading scenarios. For the model OP, it was observed in the junction of the implant-abutment complex of the dental implants during both loading scenarios. However, the average stress values recorded posteriorly were higher than anteriorly in the model TP and higher anteriorly rather than posteriorly in the model OP. Similarly, the highest value for the maximum principal stress in the bone was recorded in the crest of the bone surrounding the posterior implants. The results of the current study for the model TP matches the results of several studies performed on the All-on-4® implant supported prosthesis as that published by Kucukkurt et al. [39], Horita et al. [40], Ayali et al. [4], Moreira et al. [5], Sannino et al. [31], Lofaj et al. [36], Liu et al. [41] and Turker et al. [42, 44]. Regarding the stresses induced in the prosthetic screw, the maximum value for the Von Mises stress was recorded in the posterior screws for the model TP; matching the results of Ozan et al. [6] and Oh et al. [43]. However, for the model OP, the maximum value for the Von Mises stress was recorded in the anterior screw during both loading scenarios. Also as mentioned earlier, the anterior implants showed higher stress values than the posterior implants in the model OP. However, the difference in the average stress values between the anterior and posterior implant-abutment complexes in the model OP was less compared to the difference in the model TP. This might give a speculation for an improved load sharing between the anterior and posterior components in the model OP compared to the model TP. Such a speculation might be related to the uniform one body design of the one piece dental implant.

The higher stress value recorded posteriorly rather than anteriorly in the model TP could be attributed to the fact that the posterior implants were present in the region of load application in the group functional scenario [44]. Furthermore, distal inclination of the posterior implant was another reason mentioned by Liu et al. [41]. On the other hand, the lever arm effect and the fact that the stress value becomes more maximized as it moves further from the fulcrum may explain the reason for the maximum stress values recorded posteriorly during the canine guided scenario [13]. The higher stress values observed in the anterior implant system compared to the posterior one in the model OP could be due to a lesser bending moment of the implant framework in this model compared to the model TP thus a lesser stress was delivered to the posterior implant and more stress was delivered to the anterior one. The lesser bending moment of the implant bridge might be related to the stronger body design of the underlying one piece dental implant in addition to the short lever arm.

The canine guidance loading scenario showed less stress value compared to the group functional loading scenario in this study. Gore et al. showed similar result in their dynamic finite element analysis study comparing both occlusal schemes in implant supported fixed prostheses as well [38]. Similar findings were also reported by Turker et al. when they compared different occlusal schemes in the All-on-4® implant supported prosthesis [42, 44]. They related the lower stress values in the canine guided simulation to the anterior and posterior disocclusion of all the teeth except the canines during lateral movements of the mandible. Moreover, Abdou et al. stated in their systematic review that the group functional occlusion had double the stress values of canine guidance during lateral movements [30]. Moreover, more marginal bone loss was reported to take place in the implant supported fixed partial dentures having the group function occlusal scheme compared to those with the canine guidance occlusion. This was attributed to the greater occlusal stresses exerted in group functional occlusion. Another reason was the increased possibility of contact with the opposing teeth in the non functional lateral movements [28].

In all loading scenarios, the model TP showed higher stress values compared to the model OP regarding the implants, bone, and screws. Such a result came in line with Cehreli et al. and Hajimiragha et al. who showed lower stress values induced on the bone and the implants for the one piece dental implants in comparison to the two piece ones. This can be explained in the fact of the strong one body design and the improved mechanical properties for the one piece implant compared to the two piece one. The absence of the abutment screw contributes to such improved mechanical properties in the one-piece implant as well [14, 15, 17].

Regarding the clinical relevance of the current study results, the difference in the pattern of periimplant load distribution between both virtual models in the current study may account for the different bone remodeling that occurs clinically between them in addition to the crater like defects that appeared around the one piece dental implant. Furthermore, in the clinical setting the lower stress values recorded for the one piece monophasic dental implant may reduce the risk of component fracture; especially in the posterior region where the highest stress value was recorded in all Finite Element Analysis studies related to the All-on-4®implant supported prosthesis. Moreover, the lesser stress value recorded in the region of periimplant crestal bone in the model OP may help to reduce the rate of marginal bone loss. However, it has to be mentioned that the stress level is not the only factor that affects marginal bone loss since further factors as the design of the implant platform, presence of cantilevers, occlusal forces in addition to the implant number and diameter have their effect too [38]. Moreover, systematic reviews dealing with the issue of marginal bone loss in the one piece and two piece dental implants reported controversial results and concluded that both implant types showed no difference in their effect on marginal bone loss [16, 17]. However, such results should be held with caution due to the possible heterogeneity of the studies enrolled in the systematic reviews. The less stress values induced in the prosthetic screw in the one piece dental implants may be accompanied clinically with a reduced risk of screw loosening and thus a decreased incidence of prosthesis movement, soft tissue irritation and patient apprehension. The reduced levels of the marginal bone loss in the implant supported fixed prosthesis having canine guided occlusion reported by Koller et al. might be related to the lower stress values for a such occlusal scheme compared to the group functional one. So, the canine guided occlusion could be suggested clinically to have lesser biological and mechanical complications in the light of the results of the current study and that published by Koller et al. [28] and Abdou et al. [30]. Moreover, in the light of the current results for the dental implant design, the authors also prospect that there may be a lesser bending moment of the prosthetic superstructures when the one piece dental implant is used in the All-on-4® implant supported prosthesis.

Although standardization and variables control can be achieved in finite element analysis studies yet, this study has several limitations. Static loading was applied for simplification even though loading is dynamic during chewing functions. Moreover, it was proposed that the dental implants were completely osseointegrated with bone however, this does not simulate the natural situation. Also, the material properties of bone were linearly elastic and isotropic in this study yet, this does not come in line with consistent living tissue simulation. Furthermore, when clinically compared to the two piece dental implants, the one piece ones did not have a reduced rate of marginal bone loss and showed an increased risk of screw loosening when an intermediate abutment was used. So, the results of the current study which showed higher stress values for the two piece dental implants compared to the one piece monophasic ones do not suggest more biological and mechanical complications clinically. However, randomized clinical trials are needed to compare both implant designs regarding their biological and mechanical complications in addition to the technical limitations when used in the All-on-4® implant supported prosthesis.

## Conclusion

Within the current study limitations, the one-piece dental implants can be concluded to induce less stress compared to the two piece dental implants when used in the All-on-4® implant supported prosthesis in the different lateral occlusal schemes. Canine guided occlusion can be concluded to cause lower stress values in comparison to the group function occlusal scheme.

## Data Availability

The data of the maximum stress values are included in the published article. The dataset used for calculation of the average stress value are available from the corresponding author upon request.

## Abbreviations

STL: Standard tessellation language
SEqv: Equivalent stresses; Von-Mises stress
AP spread: Anterior–posterior spread
N: Newton
MPa: Mega Pascal
